# MiR-638 Repressed Vascular Smooth Muscle Cell Glycolysis by Targeting LDHA

**DOI:** 10.1515/med-2019-0077

**Published:** 2019-12-31

**Authors:** Shiyuan Chen, Hu Chen, Chaowen Yu, Ran Lu, Tao Song, Xiaogao Wang, Wenbo Tang, Yong Gao

**Affiliations:** 1 Department of Vascular Surgery, the First Affiliated Hospital of Bengbu Medical College, Changhuai Road 287, 233003 Bengbu City, China; 2 Department of General Surgery, the First Affiliated Hospital of Bengbu Medical College, Changhuai Road 287, 233003 Bengbu City, China

**Keywords:** VSMCs, miR-638, PDGF-bb, Proliferation, Migration and invasion, LDHA, Glycolysis

## Abstract

**Background:**

Abnormal proliferation and migration of vascular smooth muscle cells (VSMCs) accelerated vascular diseases progression, like atherosclerosis and restenosis. MicroRNAs were reported to participate in modulating diverse cellular processes. Here, we focused on exploring the role of miR-638 in VSMCs glycolysis and underlying mechanism.

**Methods:**

Cell Counting Kit-8 (CCK-8) assay was used to measure cell viability. Western blot assay was conducted to determine the expression of cell proliferation markers proliferating cell nuclear antigen (PCNA) and Ki-67, as well as Lactate dehydrogenase A (LDHA). VSMCs migration and invasion were evaluated by Transwell assay. Luciferase reporter gene assay and RNA immunoprecipitation were performed to validate the target relationship between miR-638 and LDHA. LDHA and miR-638 expression were also determined. Glycolysis of VSMCs was tested by corresponding Kits.

**Results:**

Platelet-derived growth factor-bb (PDGF-bb) promoted the VSMCs viability and down-regulated miR-638. Overexpression of miR-638 inhibited cell proliferation, migration and invasion of VSMCs. LDHA was identified as a target of miR-638, and counter-regulated by miR-638. Loss of miR-638 attenuated the suppressor effects on the proliferation, migration and invasion of VSMCs induced by LDHA down-regulation. MiR-638 inhibited the glycolysis of VSMCs by targeting LDHA.

**Conclusion:**

MiR-638 is down-regulated by PDGF-bb treatment and suppressed the glycolysis of VSMCs via targeting LDHA.

## 1 Introduction

Cardiovascular diseases, such as coronary artery disease, atherosclerosis, heart attack, are a leading cause of mortality in the world [1]. Especially the atherosclerosis, regulation and function of vascular smooth muscle cells (VSMCs) are crucial in its progression [2]. VSMCs were regarded as a necessary component of the vascular system to maintain vascular integrity [3], and the abnormal proliferation of VSMCs was suggested to be associated with the pathogenesis of cardiovascular diseases [4]. Hence, it is of great significance to elucidate the underlying molecular mechanisms.

Platelet-derived growth factor-bb (PDGF-bb), released mainly by vascular endothelial cells and platelets adjacent to vascular injury, exert its promoted effects on cells via binding to homo- and heterodimers of the two PDGF receptor (PDGFR) proteins PDGFR-α and PDGFR-β [4, 5]. Several reports have proved that introduction of PDGF-bb effectively promote cell proliferation and migration of VSMCs [6, 7].

MicroRNAs (miRNAs) are a class of non-coding single-stranded RNAs (~22 bases in length) that have potent functions in many biological processes of both pathological and physiological states [1, 8]. A growing body of evidence has demonstrated that miRNAs participate in regulating cellular processes of VSMCs, like proliferation and (or) migration and invasion through binding to the 3’-untranslated regions (3’-UTR) of target gene mRNAs, including miR-612 [6], miR-379 [7], miR-365 [9], miR-137 [10], miR-665 [11] and miR-448 [12]. In addition, miR-638, a primate-specific miRNA, was proven that plays pivotal roles in cancer development [13, 14, 15], as well as in regulation of the cell proliferation and migration in human VSMCs induced by PDGF-BB [4]. Despite great progression has been made in the molecular mechanism(s) of miR-638, little is known about its role in VSMCs proliferation, migration and invasion.

Glycolysis is a preferred metabolic pathway of cancer cells, known as Warburg effect, characterized with a high glycolytic rate that elevated uptake of glucose and transition of pyruvate to lactate, even under condition with enough oxygen [16, 17, 18, 19]. LDHA catalyzes the conversion of pyruvate to lactate in the final step of the Warburg effect and is recognized as a key point of anaerobic glycolysis [20, 21]. It has been elucidated that LDHA is essential for the proliferation and migration of VSMCs, and serves as a potential therapeutic target to prevent vessel lumen constriction during the process of atherosclerosis and restenosis [22]. High level of LDHA expression was observed in pancreatic cancer and breast cancer, suggesting that it might be a promising therapeutic target and prognostic biomarker [23, 24].

In the current study, we made efforts to clarify the effects of PDGF-bb on VSMCs and miR-638, the role of miR-638 in the proliferation, migration, invasion and glycolysis of VSMCs, as well as the potential mechanism.

## 2 Materials and methods

### 2.1 Cell culture and PDGF-bb treatment

Human aorta vascular smooth muscle cells (HA-VSMCs) were purchased from American Type Culture Collection (ATCC, Manassas, VA, USA), and the cells were cultured in Dulbecco’s Modified Eagle Medium (DMEM; Thermo Fisher Scientific, Rockford, IL, USA) supplemented with 10% fetal bovine serum (FBS; Invitrogen, Carlsbad, CA, USA) at 37°C with an atmosphere of 5% CO_2_.

VSMCs were divided into five groups, including control group (without any treatment), 1 ng/mL PDGF^‑^bb (Thermo Fisher Scientific) group, 10 ng/mL PDGF^‑^bb group, 20 ng/mL PDGF^‑^bb group and 30 ng/mL PDGF^‑^bb group, and subsequently incubated at 37˚C

### 2.2 Cell Counting Kit-8 (CCK-8) assay

After treatment with PDGF^‑^bb or transfection, VSMCs were seeded into 96-well plates at about 10,000 per well overnight, and cultured in DMEM. 10 μL CCK-8 solution (MedChem Express, Monmouth Junction, NJ, USA) was added into each well and incubated at 37°C for another 3 h. Then a spectrophotometer (xMark; Bio-Rad Laboratories, Inc., Hercules, CA, USA) was used to measure absorbance of each well at 450 nm.

### 2.3 Transfection of VSMCs

MiR-638 mimics (miR-638), miR-NC mimics (miR-NC), miR-638 inhibitors (in-miR-638), miR-NC inhibitors (in-miR-NC), si-LDHA or si-NC were obtained from (GenePharma Co. Ltd. Shanghai, China). Above oligonucleotides or plasmids were transfected into VSMCs using Lipofectamine^TM^ 2000 reagent (Invitrogen), following the manufacturer’s instructions.

### 2.4 RT-qPCR assay

MiRNAs were extracted using mirVana miRNA Isolation Kit (Life Technology, Thermo Fisher Scientific), and the qRT-PCR was performed with an all-in-one miRNA RT-qPCR Detection Kit (GeneCopoeia Inc., Rockville, MD). TRIzol reagent (Invitrogen) was used to extract total RNA from VSMCs and quantified by absorbance at 260 nm. Then 500 ng total RNA was used to produce cDNA using a PrimeScript™ RT Reagent Kit (Promega, Madison, WI, USA) following the manufacturer’s protocols. QPCR was conducted using a StepOnePlus™ Real-time PCR Systems (Applied Biosystems, Foster City, CA, USA) with SYBR^®^ Premix Ex Taq™ II (TaKaRa, Tokyo, Japan). U6 and GAPDH were used as the internal reference for miRNA and mRNA, respectively. The primers for miR-638, LDHA, U6 and GAPDH were synthesized by Invitrogen: miR-638, 5’-AGG-GATCGCGGGCGGGT-3’ (sense) and 5’-CAGTGCAGGGTC-CGAGGT-3’ (anti-sense); LDHA, 5’-TTGGTCCAGCGTAACGT-GAAC-3 (sense) and 5’-CCAGGATG-=TGTAGCCTTTGAG-3’ (anti-sense); U6, 5’-CTCGCTTCGGCAGCACATATACT-3’ (sense) and 5’-ACGCTTCACGAATTTGCGTGTC-3’ (anti-sense); GAPDH, 5’-TGCACCACCAACTGCTTAGC-3 (sense) and 5’-GGCATGGACTGTGGTCATGAG-3’ (anti-sense). All RT-qPCR reactions for each sample were performed in triplicates. The relative fold change in expression of miR-638 and LDHA with respect to a control sample were calculated using the threshold cycle 2^△△Ct^ method.

### 2.5 Western blot assay

VSMCs after different treatment lysed with Radio-Immunoprecipitation Assay (RIPA) buffer (Thermo Fisher Scientific), then the concentration of protein was monitored with a protein assay Kit (Pierce Biotechnology, Rockford, IL, USA). Equal amounts of protein lysates were loaded and separated by 10% sodium dodecyl sulfate (SDS)-polyacrylamide gels, transferred to polyvinylidene fluoride (PVDF) membranes (Millipore, Billerica, MA, USA). The membranes were firstly blocked in 5% non-fat milk for 1 h at room temperature, and incubated with primary antibodies against PCDA (1:1000 dilution), Ki-67 (1:1000 dilution), LDHA (1:1000 dilution), and GAPDH (1:2000 dilution). Then the membranes were incubated with corresponding HRP-conjugated secondary antibodies (1:2000 dilution) at room temperature for 2 h. Protein bands were visualized using an Enhanced Chemiluminescence Kit (Bio-Rad, Hercules, CA, USA), detected using ChemiDoc (Bio-Rad) and analyzed by densitometry (Image Lab, Bio-Rad).All antibodies were purchased Cell Signaling Technology (Danvers, MA, USA).

### 2.6 Transwell assay

To assess migration, 5×10^4^ VSMCs were seeded in 100 μL DMEM without FBS in the upper compartment of each chamber (8 μm pores; BD Biosciences, San Jose, CA, USA), whereas 500 μL DMEM with 10% FBS was added to the lower compartment of each chamber. After incubation for 8 h at 37°C, cells sticking to the bottom of the Transwell membrane were fixed with methanol, stained with 0.1% crystal violet (Sigma-Aldrich, St. Louis, MO, USA), then graphed and counted using an optical microscope (×100 magnification) (Olympus, Tokyo, Japan). As for invasion assay, the protocol was similar, instead, cells were seeded in the upper part of the chamber containing matrigel (BD Biosciences). All measurements were conducted in triplicate.

### 2.7 Luciferase assays

The online mirTarBase (http://mirtarbase.mbc.nctu.edu.tw/php/index.php) was used to seek the potential targets of miR-638. LDHA was identified as a downstream target of miR-638. The binding sites for miR-638 in the 3’-UTR or mutated fragment were cloned into pGL3 luciferase promoter vector (pGL3-empty, Promega) to construct luciferase reporter gene plasmid LDHA-WT or LDHA-MUT, respectively. VSMCs were seeded in 24-well plate (2×10^5^ cells per well), and co-transfected with LDHA-WT or LDHA-MUT, with or without miR-638 mimics, with or without miR-638 inhibitors using Lipofectamine^TM^ 2000 reagent according to the manufacturer’s instructions. Cells were harvested after transfection for 48 h and a Dual-Luciferase Reporter Assay System (Promega) was used to detect luciferase activities. Each experiment was repeated three times.

### 2.8 RNA immunoprecipitation (RIP)

RIP was conducted according to the manufacturer’s instructions using EZ-Magna RIP Kit (Millipore). VSMCs were harvested and lysed in RIP lysis buffer, followed by incubation of protein A/G magnetic beads with human anti-Argonaute2 (Ago2) antibody (Abcam, Cambridge, MA, USA) or IgG (Cell Signaling Technology, Danvers, MA, USA). Then RNAs in magnetic beads-binding complexes were purified by digestion with proteinase K. Finally, RT-qPCR assay was performed to detect enrichment patterns of miR-638 and LDHA.

### 2.9 LDHA activity

LDHA activity was detected by measuring the reduction of NADH in the reaction system consist of 20 mM HEPES (pH 7.2), 20 μM NADH, 0.05% bovine serum albumin, and 2 mM pyruvate using microplate reader (excitation,340 nm; emission, 460 nm) as previous described [21].

### 2.10 Measurement of glucose consumption and lactate production

The glucose consumption and lactate production were measured using the Glucose Uptake Assay Kit (Colorimetric, Abcam) and Lactate Assay Kit (Sigma-Aldrich) referring to the protocols of manufacturer, respectively.

### 2.11 Statistical analysis

Analysis of results was performed using GraphPad Prism (GraphPad Software, San Diego, CA, USA). All data were exhibited as mean ± SD (standard deviation). Student’s t-test or one-way variance analysis was used to analyze difference of data in various groups. *P*<0.05 was considered to be statistically significant.

## 3 Results

### 3.1 PDGF-bb promoted the cell viability of VSMCs and down-regulated miR-638 expression

**Figure 1:**
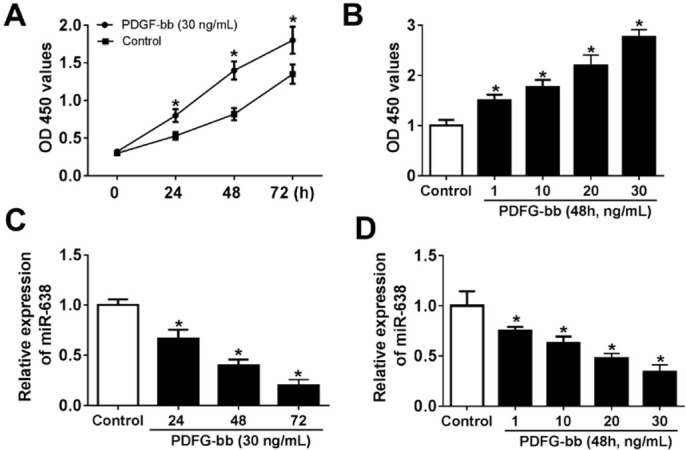
PDGF-bb ptomoted the cell viability of VSMCs and down-regulated miR-638 expression. (A and C) The cell viability and miR-638 level of VSMCs at 24 h, 48 h and 72 h after treatment with 30 ng/mL PDGF-bb. (B and D) The cell viability and miR-638 level of VSMCs at 48 h after treatment with PDGF-bb at concentrations of 1 ng/mL, 10 ng/mL, 20 ng/mL or 30 ng/mL. Each experiment was performed in triplicate. *P<0.05, control (without treatment).

CCK-8 assay was used to clarify whether PDGF-bb could elevate the proliferation viability of VSMCs, and RT-qPCR assay was carried out to evaluate the expression level of miR-638. The cell viability of VSMCs showed an obvious augment after treatment with 30 ng/mL PDGF-bb, compared with that in control group, and the cell viability increased in a time-dependent manner (Fig. 1A). As shown in Fig. 1B, the promotion of cell viability of VSMCs induced by different concentrations of PDGF-bb also exhibited concentration-dependence. Compared with control group, the expression of miR-638 significantly decreased in VSMCs treated with 30 ng/mL PDGF-bb, and exhibited time-dependence (Fig. 1C). In addition, PDGF-bb inhibited the level of miR-638 in a concentration-dependent manner (Fig. 1D).

### 3.2 Up-regulation of miR-638 suppressed cell proliferation, migration and invasion of VSMCs *in vitro*

**Figure 2:**
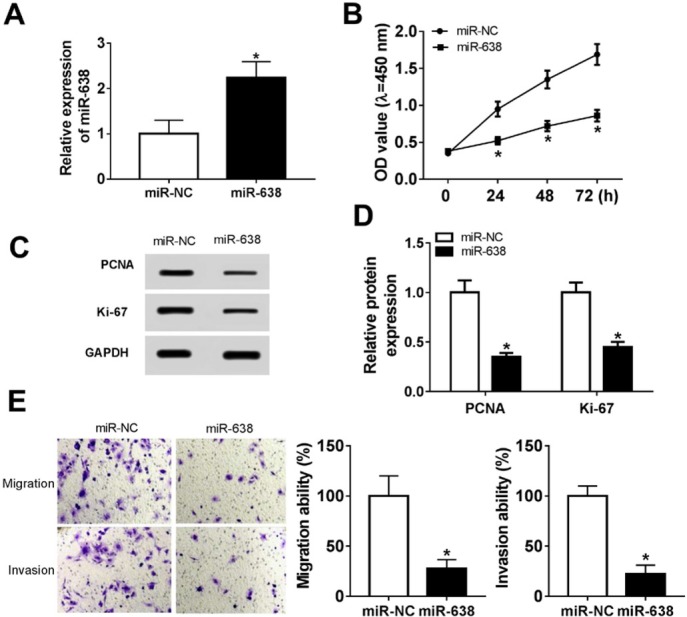
Up-regulation of miR-638 suppressed cell proliferation, migration and invasion of VSMCs in vitro. VSMCs were transfected with miR-638 mimic or miR-NC mimic (A) The mRNA expression level of miR-638 of VSMCs after transfection was measured by RT-qPCR assay. (B) The cell viability of VSMCs was tested via CCK-8 assay at 24 h, 48 h and 72 h post transfection. (C and D) The protein levels of PCDA and Ki-67 in VSMCs were evaluated by western blot assay after transfection. (E) The migration and invasion abilities was detected through Transwell assay. Each experiment was performed in triplicate. *P<0.05.

To figure out the role of miR-638 in the progression of VSMCs, we transfected VSMCs with miR-638 mimic or miR-NC mimic, and then we found that the level of miR-638 was up-regulated after transfection with miR-638 mimic (Fig. 2A). CCK-8 assay was conducted to detect the effect of the up-regulation of miR-638 on the cell viability of VSMCs, and indicated that the cell viability was significantly inhibited by miR-638 mimic (Fig. 2B). Western blot assay results showed the protein levels of cell proliferation markers PCNA and Ki-67 strikingly decreased in VSMCs transfected with miR-638 mimic (Fig. 2C-2D). Following Transwell assay indicated that overexpression of miR-638 effectively impeded the migration and invasion abilities of VSMCs (Fig. 2E).

### 3.3 LDHA was a downstream target of miR-638 and negatively regulated by miR-638

**Figure 3:**
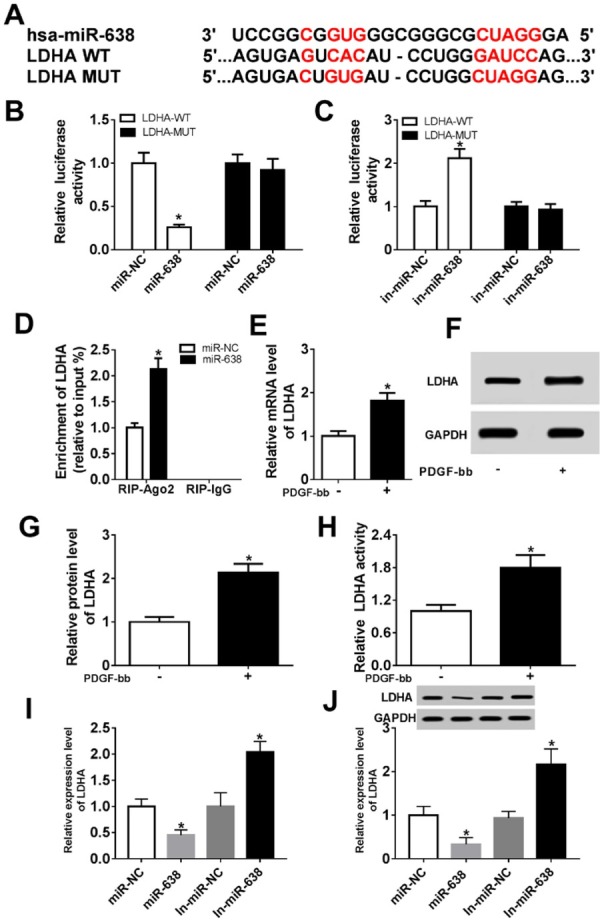
LDHA was a downstream target of miR-638 and negatively regulated by miR-638 (A) The putative binding site and the mutant sites of miR-638 on the 3’-UTR of LDHA mRNA are marked in red. (B) VSMCs were co-transfected with LDHA-WT or LDHA-MUT and miR-638 mimics or miR-NC mimics, followed by the detection of luciferase activities at 48 h after transfection. (C) VSMCs were co-transfected with LDHA-WT or LDHA-MUT and miR-638 inhibitors or miR-NC inhibitors, followed by the detection of luciferase activities at 48 h after transfection. (D) RIP was conducted, and expression of LDHA was determined. (E-G) The mRNA level and protein level of LDHA in VSMCs were tested by RT-qPCR and western blot assay treatment with PDGF-bb. (H) LDHA activity in PDGF-treated VSMCs. (I) Thd mRNA level of LDHA in VSMCs transfected with miR-NC mimics, miR-638mimics, miR-NC inhibitors or miR-638 inhibitors was evaluated by RT-qPCR. *P<0.05. (J) The protein level of LDHA was determined by western blot assay. Each experiment was performed in triplicate.*P<0.05.

The mirTarBase website was used to predict the potential targets of miR-638. Then we found that the miR-638 could bind with the 3’-UTR of LDHA (Fig. 3A). Subsequent luciferase reporter assay was employed to further confirm the interaction between miR-638 and LDHA. Luciferase activity results demonstrated that the luciferase activity of VSMCs co-transfected with LDHA-WT and miR-638 mimics was strikingly decreased (Fig. 3B), while in cells co-transfected with LDHA-WT and in-miR-638, luciferase activity was apparently elevated (Fig. 3C). No obvious change was observed in the luciferase activity of cells co-transfected with LDHA-MUT and miR-638 mimics or in-miR-638 (Fig. 3B-3C). Following RIP further proved the target relationship between miR-638 and LDHA (Fig. 3D). Notably, the mRNA level of LDHA was increased after treatment with PDGF-bb (Fig. 3E). Additionally, PDGF-bb treatment also enhanced protein level of LDHA (Fig. 3F-3G). Moreover, LDHA activity was elevated in PDGF-bb-treated VSMCs (Fig. 3H). RT-qPCR assay indicated that up-regulated miR-638 inhibited the mRNA level of LDHA, while down-regulated miR-638 induced opposite effect (Fig. 3I). The protein level of LDHA was measured by western blot assay, the results then suggested that LDHA expression was negatively regulated by miR-638 (Fig. 3J).

### 3.4 Silence of miR-638 partially reversed knock-down of LDHA-mediated suppression on VSMCs proliferation, migration and invasion

**Figure 4:**
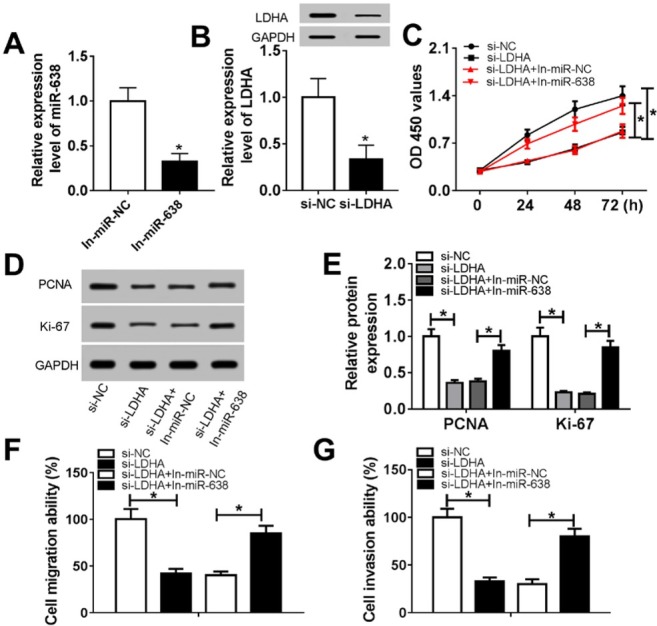
Silence of miR-638 partially reversed knock-down of LDHA-mediated supepression on VSMCs proliferation, migration and invasion (A) The level of miR-638 after transfection with miR-638 inhibitors. (B) The protein level of LDHA after transfection with si-LDHA. (C) The cell ability of VSMCs after co-transfection. (D-E) The protein level of PCDA and Ki-67 after co-transfection. (F-G) The number of migration and invasion VSMCs after co-transfection. Each experiment was performed in triplicate.*P<0.05.

To further investigate the mechanism of miR-638 on progress of VSMCs, we constructed VSMCs that LDHA down-regulated or with miR-638 silenced meanwhile. As shown in Fig. 4A, the expression level of miR-638 remarkably decreased after transfection with miR-638 inhibitors. Western blot assay indicated that the level of LDHA notably inhibited by si-LDHA (Fig. 4B). CCK-8 assay and western blot assay suggested that knock-down of LDHA suppressed the cell viability of VSMCs and proliferation marker protein levels of VSMCs, while silence of miR-638 could weaken the suppressor effects on the VSMCs proliferation triggered by the loss of LDHA (Fig. 4C-4E). Significant repression in migration and invasion abilities of VSMCs treated with si-LDHA was observed through Transwell assay, silence of miR-638 attenuated knock-down of LDHA-mediated suppression on the migration and invasion abilities of VSMCs (Fig. 4F-4G).

### 3.5 MiR-638 inhibited the glycolysis of VSMCs by target LDHA

**Figure 5:**
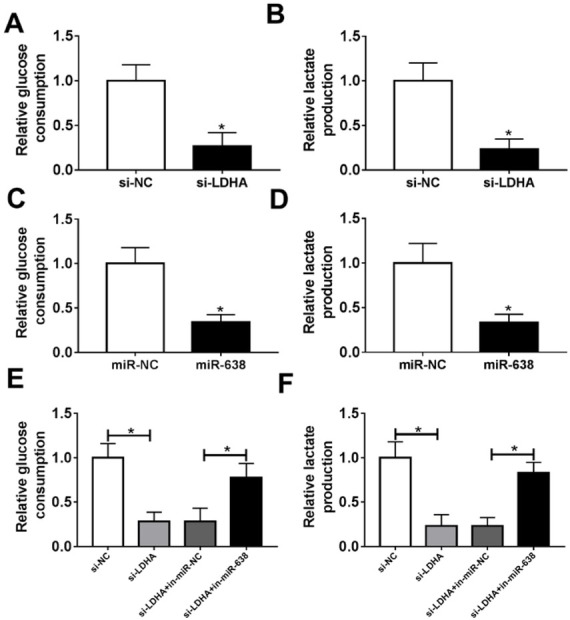
miR-638 inhibited the glycolysis of VSMCs by target LDHA. (A and B) VSMCs were transfected with si-LDHA or si-NC. The glucose consumption and lactate production of the cells were evaluated. (C and D) VSMCs were transfected with miR-638 mimic or miR-NC mimics. The glucose consumption and lactate production of the cells were detected. (E-F) VSMCs were transfected with si-NC, si-LDHA, si-LDHA + miR-NC mimic or si-LDHA + miR-638 mimic. The glucose consumption and lactate production of the cells were analyzed. Each experiment was performed in triplicate. *P<0.05.

To further investigate the roles of LDHA and miR-638 in glycolysis, we measured the glucose consumption and lactate production in VSMCs after transfection with si-LDHA or miR-638 mimic as well as corresponding negative control. Knock-down of LDHA caused a notable reduction of the glucose consumption and lactate production (Fig. 5A-5B). Introduction of miR-638 also triggered an evident decrease of the glucose consumption and lactate production in VSMCs (Fig. 5C-5D). As shown in Fig. 5E-5F, Silenced miR-638 almost reversed knock-down of LDHA-mediated decrease of glucose consumption and lactate production. These results indicated that miR-638 may regulate glycolysis by modulating LDHA.

## 4 Discussion

Vascular smooth muscle cells (VSMCs) compose the major structure of vascular system, and exert functions such like maintaining vessel tone and blood pressure [25]. Therefore, a better understanding of the VSMCs progression will support theoretical basis for promoting potent therapeutic approaches for cardiovascular diseases.

PDGF-bb was the most efficient stimulants among PDGF family for the VSMC proliferation and migration by regulating several transcription factors and key molecular signaling pathways [4, 5]. Chen *et al*. demonstrated that treatment with PDGF-bb for 6 h significantly promoted the proliferation and migration of VSMCs [6]. In addition, Li and his colleagues found that PDGF-bb promotes the cell proliferation of VSMCs, which determined by CCK-8 assay [7]. Zhang *et al*. also detected the promoted role of PDGF-bb on VSMCs proliferation [12]. In our study, we found that PDGF-bb promoted the cell ability of VSMCs and suppressed miR-638 expression in a concentration-and time-dependent manner, which in accordance with former studies.

Previous reports indicated that the VSMCs proliferation, migration and invasion could be regulated by miRNAs. Chen *et al*. demonstrated that miR-612 inhibited VSMCs proliferation and migration through its direct modulation on AKT2 [6]. MiR-379 inhibits the proliferative, invasive, and migratory abilities of VSMCs by targeting insulin-like factor-1 [7]. The study performed by Zhang *et al*. suggested an anti-proliferative role for miR-365 in VSMC proliferation [9]. The repressive effects of miR-137 on cell proliferation and migration of VSMCs was validated [10]. And miR-665 suppressed the VSMCs proliferation, invasion and migration via directly targeting FGF9 and MEF2D [11]. On the contrary, up-regulation of miR-448 accelerated the proliferation and migration of VSMCs [12]. As for miR-638, researchers found its tumor inhibitor role in human gastric carcinoma, colorectal carcinoma and osteosarcoma [13, 14, 15]. In the current study, we verified that miR-638 showed a suppressive character in VSMCs proliferation, migration and invasion *in vitro*.

Despite the conclusion has been made that miR-638 modulated human VSMCs proliferation and migration via regulating the NOR1/cyclin D pathway [4], we tried to seek other potential mechanism(s). Bioinformatics analysis results suggested that LDHA was a target of miR-638, and following luciferase reporter assay and RIP further confirmed the interaction. LDHA is a growth factor and has been proved to regulate cell proliferation and migration of VSMCs [22]

Several publications have demonstrated that LDHA are regulated by the miRNAs [26, 27, 28]. Among these, LDHA promoted glycolysis and cell proliferation of breast cancer MCF-7 and MDA-MB-231 cells, and its beneficial effects on glycolysis and cell proliferation can be impaired by miR-34a [26]. MiR-383 functioned as an inhibitor on the proliferation of ovarian cancer cell by targeting LDHA and suppressing the glycolysis [27]. A previous study showed that miR-323a-3p inhibited the growth of Osteosarcoma (OS) cell by targeting LDHA and suppressed the glycolysis of OS [28]. These results indicate that LDHA is a potential target of miRNAs to modulate the glucose metabolism of cancers. In our study, we also validated that LDHA is a downstream target of miR-638, and negatively regulated by miR-638, while positively regulated by PDGF-bb. Additionally, we observed that depletion of LDHA suppressed the proliferation, migration, invasion and glycolysis of VSMCs, while the suppressive effects could be attenuated by knock-down of miR-638, suggesting that miR-638 suppressed the proliferation, migration, invasion and glycolysis of VSMCs via directly targeting LDHA.

In summary, we found the enhancement effects of PDGF-bb on the cell viability of VSMCs and suppressive effect on the expression level of miR-638. We also observed that the up-regulation of miR-638 inhibited the proliferation, migration and invasion of VSMCs. Then LDHA was identified as a direct target of miR-638 via bioinformatics analysis online, which further confirmed by following Luciferase reporter assay and RIP. PDGF-bb treatment effectively facilitated the LDHA expression and LDHA activity. In addition, LDHA expression was negatively regulated by miR-638. Depletion of LDHA also inhibited the proliferation, migration and invasion abilities of VSMCs, while simultaneous silence of miR-638 weakened the suppressor effects in part. What’s more, miR-638 inhibited the glycolysis of VSMCs by target LDHA. In short, the present study indicated that miR-638 could repress the proliferation, migration, invasion abilities and glycolysis of VSMCs by targeting, at least in part, LDHA, which provides a novel potential therapeutic strategy for human vascular diseases, such as atherosclerosis and restenosis.
